# Study on the Micro-Nano Characteristics of Organic-Rich Shale Reservoirs Under Differential Sedimentation: A Case Study of the Lower Silurian Longmaxi Formation and Upper Permian Dalong Formation Shales in the Sichuan Basin, China

**DOI:** 10.3390/nano16070440

**Published:** 2026-04-03

**Authors:** Jia Wang, Sirui Liu, Tao Wang, Tianzhu Hu, Qi Zhang, Mingkai Zhang, Xinrui Yang, Dunfan Wang

**Affiliations:** 1 School of Petroleum Engineering, Chongqing University of Science and Technology, Chongqing 401331, China; 2 Chongqing Key Laboratory of Complex Oil and Gas Exploration and Development, Chongqing University of Science and Technology, Chongqing 401331, China; 3 Chongqing Gas Field, PetroChina Southwest Oil and Gas Field Company, Chongqing 401120, China

**Keywords:** reservoir, nanopores, organic matter, Longmaxi Formation, Dalong Formation, Sichuan Basin

## Abstract

Both the Lower Silurian Longmaxi Formation and the Upper Permian Dalong Formation shales in southern China are organic-rich with well-developed nanoscale reservoir pores, demonstrating significant shale gas exploration potential. However, the current lack of in-depth research on the differential depositional and reservoir evolution characteristics of these two shale sequences has left the main controlling factors of the reservoirs unclear, thereby constraining breakthroughs in shale gas development. Focusing on the Longmaxi and Dalong formation shales in the Sichuan Basin, this study employed various analytical methods, including major and trace element analyses, X-ray diffraction (XRD), high-pressure mercury intrusion (HPMI), nitrogen adsorption, CO_2_ adsorption, and scanning electron microscopy (SEM). Investigations into the depositional paleoenvironment, paleoproductivity, organic matter enrichment, and microscopic difference mechanisms of nanoscale reservoirs reveal that the Longmaxi Formation shale represents a passive continental margin shelf facies. It is characterized by strong terrigenous input, a predominance of quartz and clay minerals, and consists mainly of siliceous and argillaceous shale facies with high organic matter abundance. In contrast, the Dalong Formation shale was deposited in an intra-platform basin under the influence of intra-platform rifting. It features weak terrigenous input, highly reducing conditions, and strong paleoproductivity. Dominated by quartz and carbonate minerals, its lithofacies are primarily siliceous and calcareous shales. Within the Dalong Formation, the diagenetic dissolution of carbonate minerals promotes the development of micrometer-scale pores larger than 100 μm, while the extensive thermal evolution of organic matter fosters the formation of honeycomb- and embayment-like nanoscale micropores and mesopores, rendering it a relatively superior shale reservoir. Ultimately, the high-TOC shales in the lower part of the Longmaxi Formation and the upper part of the Dalong Formation are identified as the primary sweet spot intervals for future shale gas development.

## 1. Introduction

Shale gas, an emerging clean energy resource, has attracted immense global attention over the past two decades [1]. Following successful exploration and development in China, the Lower Silurian Longmaxi Formation in the southern Sichuan Basin has emerged as the country’s first hundred-billion-cubic-meter shale gas field, with cumulative production exceeding 100 billion cubic meters [2]. In recent years, wells deployed by Sinopec in the Chengkou-Exi Trough (e.g., wells HY1, Leiye 1, and Mao 1) and by PetroChina in the Kaijiang-Liangping area (e.g., wells Daye 1H, D201, and D202) have successively obtained 6.4~42.66 × 10^4^ m^3^/d commercial gas flows from the Upper Permian Wujiaping and Dalong formations [3]. These achievements demonstrate the significant exploration and development potential of both Silurian and Permian marine shales in the Sichuan Basin [4]. The effective enrichment of shale gas is comprehensively controlled by factors such as shale hydrocarbon generation potential and reservoir quality. Although scholars have conducted extensive research on the hydrocarbon generation potential and reservoir evolution processes of organic-rich marine shales in southern China [5,6], in-depth studies on the differential patterns of shale mineral composition, organic geochemical characteristics, and reservoir pore types and structures under varying depositional conditions remain insufficient [7,8]. What is more, there is no comprehensive comparative study on the sedimentary-reservoir characteristics of marine shales from different horizons. This knowledge gap has impeded further breakthroughs in shale gas exploration and development. Taking the organic-rich shales of the Lower Silurian Longmaxi Formation in the central Sichuan Basin and the Upper Permian Dalong Formation in the northeastern Sichuan Basin as examples, this study utilizes advanced experimental techniques for microscopic reservoir characterization, including major and trace element analyses, field emission scanning electron microscopy (FE-SEM), low-temperature nitrogen adsorption, and CO_2_ adsorption. By investigating the depositional paleoenvironment, paleoproductivity, and microscopic reservoir characteristics, this research ultimately reveals the differential mechanisms of shale deposition and reservoir evolution under distinct depositional conditions. The findings aim to provide a new theoretical basis for the optimization of favorable target areas and well placement for marine shale gas development in China.

## 2. Regional Geological Setting

**Figure 1:**
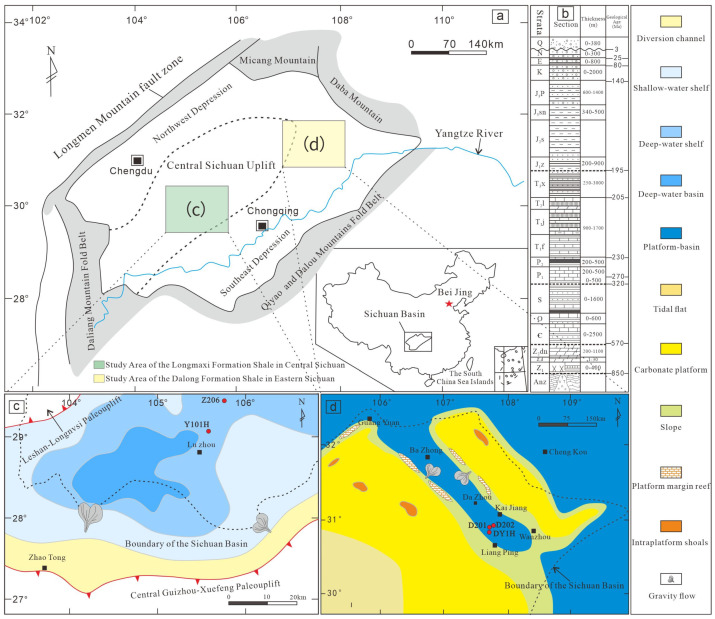
Stratigraphic table of the Sichuan Basin and map of sedimentary facies. (**a**) Location Map of the Sichuan Basin. (**b**) Stratigraphic Table of the Sichuan Basin. (**c**) Sedimentary Facies Map of the Longmaxi Formation in Southern Sichuan; (**d**) Sedimentary Facies Map of the Dalong Formation in Northeastern Sichuan.

Located in southwestern China, the Sichuan Basin is a typical superimposed petroliferous basin characterized by multiple source-reservoir-caprock assemblages. It serves as a core producing area for both conventional natural gas and shale gas in China, with cumulative gas production reaching up to 800 billion cubic meters [9,10]. Situated on the western margin of the Yangtze Plate, the basin’s tectonic framework is primarily composed of the basin itself and its peripheral orogenic belts, including the Longmenshan, Micangshan-Dabashan, Qiyaoshan, Daloushan, and Daliangshan fold-thrust belts. Multi-stage and diverse tectonic transition processes and complex structural interactions between the basin and these orogenic belts have formed an organically connected, composite basin-mountain system (Figure 1a). Although the Sichuan Basin has experienced varying degrees of uplift and denudation under the influence of multi-stage tectonic movements—resulting in the widespread absence of Devonian and Carboniferous strata—it nonetheless features extensive deposition of fine-grained shelf facies within the Lower Silurian Longmaxi Formation, with thicknesses ranging from 400 to 1300 m, High-quality shales are mainly distributed at the bottom of the Lower Silurian, with a general thickness of less than 100 m (Figure 1b). Furthermore, intra-platform basin shale deposits of the Upper Permian Dalong Formation are widely developed in the eastern Sichuan region (Figure 1b), with thicknesses generally between 20 and 40 m. Ultimately, these two stratigraphic sequences serve not only as high-quality source rocks but also as the primary target strata for current shale gas exploration and development in the Sichuan Basin.

Since the Late Ordovician, driven by the continuous uplift of the Leshan-Longnüsi paleo-uplift on the western margin of the Sichuan Basin and the Qianzhong-Xuefeng paleo-uplift, the Upper Yangtze Craton basin experienced a reduction in its spatial extent. Coinciding with a global marine transgression during the deposition of the Early Silurian Longmaxi Formation, the formerly expansive open-marine region transformed into a restricted marine environment characterized by a paleogeographic framework of “three uplifts flanking one depression” [11]. In Luzhou and its western adjacent areas, the depositional water mass was relatively deep, representing a typical deep-water shelf environment (Figure 1c). Lithologically, the Longmaxi Formation is dominated by siliceous shale, argillaceous siltstone, silty shale, and siltstone, exhibiting a distinct upward-coarsening grain size sequence. Its depositional thickness ranges from 500 to 650 m, with present-day burial depths varying between 3500 and 4500 m [5,12].

During the Late Permian, the entire Sichuan Basin was profoundly impacted by the Dongwu Movement and the expansion of the Mianlue Ocean. The southwestern region was predominantly characterized by the uplift of the Emeishan superplume, whereas in the northeast, the Mianlue oceanic crust subducted beneath the Qinling Block [13]. Consequently, the paleobathymetry exhibited a gradual deepening trend from the southwest to the northeast. Concurrently, the NW-SE trending, U-shaped Kaijiang-Liangping Trough—opening towards both Guangyuan-Wangcang and Liangping—was formed in the northeastern part of the basin (Figure 1d). The Late Permian strata in the northeastern Sichuan Basin display pronounced lateral facies transitions. Within the trough, deep-water, fine-grained deposits of the Dalong Formation’s intra-platform basin facies were accumulated. Conversely, the carbonate platform and slope facies of the Changxing Formation developed along both flanks of the trough [7,14]. Lithologically, the Dalong Formation within the trough is dominated by calcareous and siliceous shales, with a depositional thickness of less than 40 m and present-day burial depths ranging from 4000 to 4500 m [15].

## 3. Experimental Methods

To achieve the research objectives, a total of 54 Longmaxi Formation shale samples from wells Z206 and Y101 in the central Sichuan Basin, along with 47 Dalong Formation shale samples from wells Daye 1H and D201 in the northeastern Sichuan Basin, were selected for whole-rock X-ray diffraction (XRD) and total organic carbon (TOC) analyses. Among these, 14 Longmaxi samples from Well Z206 and 16 Dalong samples from Well Daye 1H were subjected to major and trace element testing. Additionally, routine porosity and permeability analyses, as well as scanning electron microscopy (SEM) observations, were performed on 47 Longmaxi Formation samples from Well Z206 and 30 Dalong Formation massive shale samples from Well Daye 1H. Concurrently, a targeted subset of these—5 Longmaxi samples from Well Z206 and 9 Dalong massive samples from Well Daye 1H—was selected for HPMI, low-temperature nitrogen adsorption, and CO_2_ adsorption experiments. All aforementioned experiments were conducted at the State Key Laboratory of Oil and Gas Reservoir Geology and Exploitation and the Chongqing Key Laboratory of Complex Oil and Gas Field Exploration and Development. The experimental procedures strictly adhered to the petroleum and natural gas industry standards of the People’s Republic of China (GB/T 14505-1993, GB/T 34533-2023, GA/T 2079-2023, NB/T 14008-2015, DZ/T 0455-2023) [16,17,18,19,20]. During the sample preparation and testing phase, no less than 100 g of shale particles were first ground to under 200 mesh for whole-rock XRD analysis using a Rigaku SmartLab SE X-ray Diffractometer (Rigaku Corporation, Tokyo, Japan). Following Soxhlet extraction, TOC was determined utilizing a Shimadzu TOC-L analyzer (Shimadzu Corporation, Kyoto, Japan). After acidification and digestion, major and trace element concentrations were quantified using an Expec 7000 (Focused Photonics Inc., FPI, Hangzhou, China) inductively coupled plasma mass spectrometer (ICP-MS). Subsequently, selected shale particles were observed via a JEOL JSM-7800F field emission scanning electron microscope (JEOL Ltd., Tokyo, Japan). For the low-temperature nitrogen adsorption experiments, the shale was ground to under 100 mesh and analyzed using a Quantachrome Quadrasorb SI instrument (Quantachrome Instruments, Boynton Beach, FL, USA). Finally, small cylindrical core plugs (2.5 cm in diameter and >5 cm in length) were drilled for routine porosity and permeability measurements. Following these tests, the same cylindrical plugs were subjected to HPMI analysis using a Micromeritics AutoPore IV 9510 automated mercury porosimeter (Micromeritics Instrument Corporation, Norcross, GA, USA).

## 4. Results

### 4.1. Petrological Characteristics

**Figure 2:**
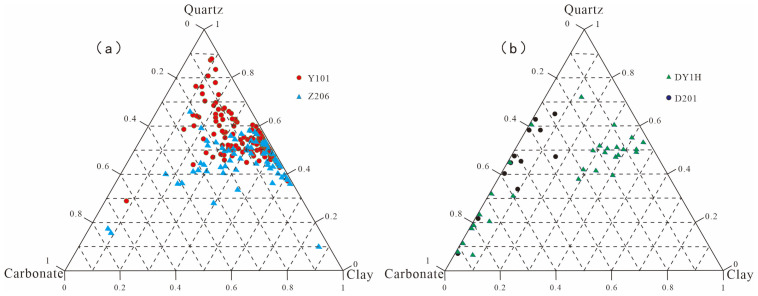
Ternary diagram of shale mineral composition. (**a**) Ternary diagram showing mineral compositions of Longmaxi Formation shale. (**b**) Ternary diagram showing mineral compositions of Dalong Formation shale.

XRD data indicate that the Longmaxi Formation shale is predominantly composed of quartz and clay minerals. Specifically, the quartz content ranges from 10% to 88% with an average of 51.31%. Clay minerals range from 6% to 87%, averaging 35.33%, while carbonate minerals range from 0% to 76%, with a mean value of 10.58%. In comparison, the Dalong Formation shale mainly consists of quartz, carbonate, and clay minerals. Quartz remains the most abundant constituent, varying between 3% and 91% with an average of 42.49%. This is followed by carbonate minerals, which range from 2% to 96% (averaging 38.20%). Clay minerals exhibit the lowest overall average content at 12.75%, despite a wide compositional range from 3% to 91%. According to the three-end-member ternary classification scheme for shales, the Longmaxi Formation is primarily classified into siliceous shale and argillaceous shale facies (Figure 2a). Conversely, the Dalong Formation is predominantly characterized by calcareous shale and calcareous-siliceous shale facies (Figure 2b).

### 4.2. Geochemical Characteristics

#### 4.2.1. Organic Geochemical Characteristics

The TOC contents of the 54 Longmaxi Formation shale samples range from 0.43% to 4.73% (average: 2.21%). In comparison, the 47 Dalong Formation shale samples exhibit generally higher TOC values, ranging from 0.40% to 11.88% (average: 4.35%). Although both the Lower Silurian Longmaxi and the Upper Permian Dalong formation shales qualify as high-quality source rocks, the Dalong Formation shale demonstrates a distinctly stronger hydrocarbon generation potential.

#### 4.2.2. Inorganic Geochemical Characteristics

Analytical results for major and trace elements in 14 Longmaxi Formation shale samples indicate significant variations in major element compositions. Major elements of Longmaxi Formation shale mainly include SiO_2_ (15.80 wt.%), Al_2_O_3_ (61.87 wt.%), Fe_2_O_3_ (4.20 wt.%), and K_2_O (3.12 wt.%). Trace element concentrations range from 0.82 to 3725.72 ppm, with the most abundant being Ba (2469.21 ppm), Pb (27.77 ppm), Cs (10.90 ppm), Mo (9.73 ppm), and U (9.62 ppm). Conversely, the analysis of 16 Dalong Formation shale samples reveals that major element contents are primarily composed of SiO_2_ (50.85 wt.%), CaO (15.47 wt.%), Al_2_O_3_ (7.38 wt.%), and Fe_2_O_3_ (2.86 wt.%). The trace element assemblages in the Dalong Formation are dominated by Sr (718.4 ppm), Zr (148.5 ppm), Cr (149.3 ppm), Zn (114.0 ppm), and Cu (57.8 ppm).

### 4.3. Reservoir Microscopic Characteristics

**Figure 3:**
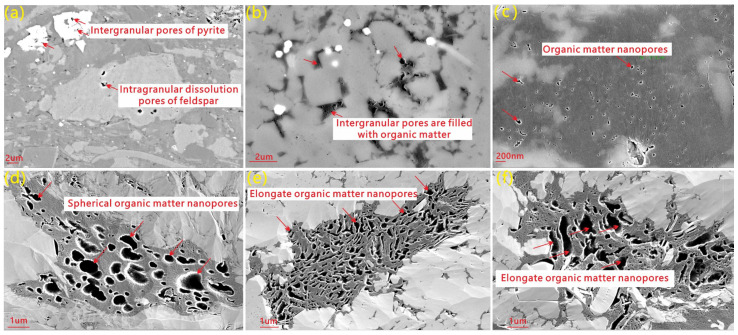
Plate of reservoir microscopic structural characteristics where the arrows point. (**a**) Well DY1-1H, 4328.1 m, Dalong Formation, intergranular pores of pyrite and intragranular dissolution pores of feldspar. (**b**) Well Z206, 4263.98 m, Longamxi Formation, Intergranular pores are filled with organic matter; (**c**) Well Z206, 4277.36 m, Longamxi Formation, organic matter nanopores; (**d**) Well DY1-1H, 4328.1 m, 4336.17 m, Dalong Formation, spherical organic matter nanopores in the kerogen; (**e**) Well DY1-1H, 4364.93 m, Dalong Formation, elongate organic matter nanopores in the Bitumen; (**f**) Well Z206, 4241.46 m, Longamxi Formation, elongate organic matter nanopores in the Bitumen.

Both the Longmaxi and Dalong formation shales are characterized as “ultra-low porosity and ultra-low permeability” reservoirs. For the Longmaxi Formation shale, porosity ranges from 0.83 to 5.44 vol.% (average: 3.02 vol.%), with permeability ranging from 0.000033 to 0.007 mD (average, 0.0012 mD). In comparison, the Dalong Formation exhibits slightly better petrophysical properties, with porosity ranging from 2.21 to 6.36 vol.% (average, 3.65 vol.%) and permeability varying between 0.013 and 0.076 mD (average: 0.028 mD). In both formations, the pore systems are dominated by intragranular dissolution pores within quartz, feldspar, and calcite (Figure 3a), intercrystalline pores (Figure 3b), organic matter (OM) pores (Figure 3c–f), and microfractures. Notably, the OM pores primarily exhibit two distinct morphological types: honeycomb-like and embayment-like. The honeycomb-like OM pores are mainly distributed within kerogen (Figure 3c,d), whereas the embayment-like OM pores predominantly occur within the bitumen that fills intergranular pores (Figure 3e,f).

#### 4.3.1. High-Pressure Mercury Intrusion Porosimetry

**Figure 4:**
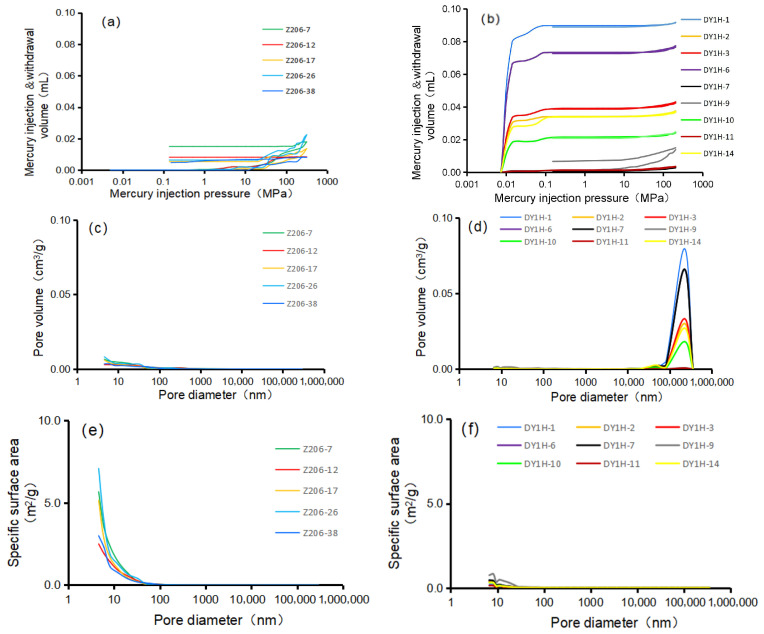
Plates of experimental data for shale High-Pressure Mercury Intrusion. (**a**) Variation of mercury intrusion and extrusion volumes for Longmaxi Formation Shale under High-Pressure Mercury Intrusion at different pressures. (**b**) Variation of mercury intrusion and extrusion volumes for Dalong Formation Shale under High-Pressure Mercury Intrusion at different pressures. (**c**) Pore volume of different pore sizes per unit mass of Longmaxi Formation Shale. (**d**) Pore volume of different pore sizes per unit mass of Dalong Formation Shale. (**e**) Specific surface area of different pore sizes per unit mass of Longmaxi Formation Shale. (**f**) Specific surface area of different pore sizes per unit mass of Dalong Formation Shale.

HPMI data obtained from 5 Longmaxi Formation shale samples (Well Z206) and 9 Dalong Formation shale samples (Well Daye 1H) reveal notable differences in pore structures. The cumulative mercury intrusion volume for the Longmaxi Formation shale ranges from 0.00816 to 0.02246 mL (average: 0.0141 mL). In comparison, the Dalong Formation shale exhibits a wider range, from 0.00316 to 0.09140 mL (average: 0.0350 mL). Under the same pressure conditions, the mercury intrusion volume of the Dalong Formation is significantly higher than that of the Longmaxi Formation (Figure 4a,b). Furthermore, the Longmaxi Formation shale is predominantly characterized by the development of nanoscale pores, with pore diameters mostly less than 100 nm (Figure 4c). Conversely, pore diameters in the Dalong Formation shale are primarily distributed in the macropore to microfracture range of 100,000 to 300,000 nm (Figure 4d). Consequently, an inverse relationship is observed in their specific surface areas. Driven by the extensive development of nanopores, the Longmaxi Formation exhibits a relatively large specific surface area, ranging from 2.493 to 7.080 m^2^/g (average: 4.674 m^2^/g) (Figure 4e). On the contrary, dominated by much larger pores, the Dalong Formation shale possesses a significantly smaller specific surface area, mainly distributed between 0.137 and 0.748 m^2^/g (average: 0.331 m^2^/g) (Figure 4f).

#### 4.3.2. Nitrogen Adsorption Experiment

**Figure 5:**
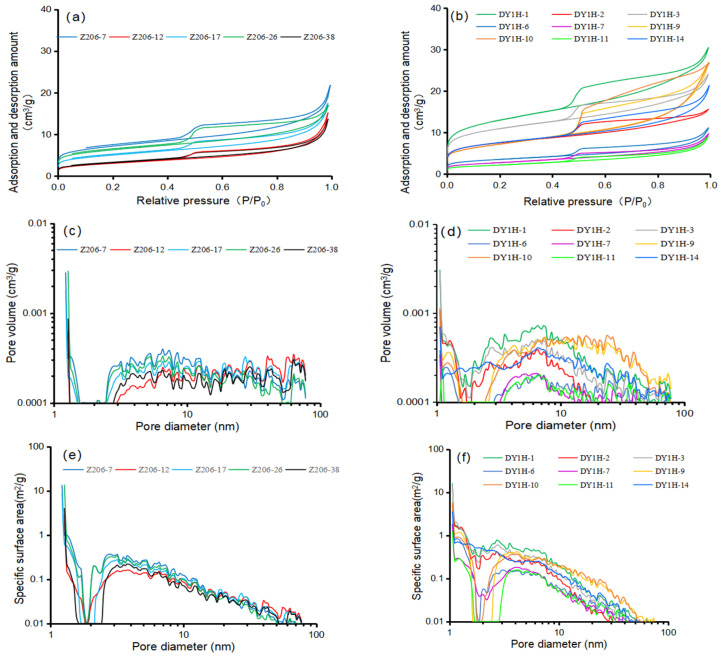
Plates of experimental data for shale Nitrogen Adsorption. (**a**) Adsorption and desorption volumes per unit mass of Longmaxi Formation Shale under different pressure conditions. (**b**) Adsorption and desorption volumes per unit mass of Dalong Formation Shale under different pressure conditions. (**c**) Pore volume of different pore sizes per unit mass of Longmaxi Formation Shale. (**d**) Pore volume of different pore sizes per unit mass of Dalong Formation Shale. (**e**) Specific surface area of different pore sizes per unit mass of Longmaxi Formation Shale. (**f**) Specific surface area of different pore sizes per unit mass of Dalong Formation Shale.

The same 5 shale samples from the Longmaxi Formation and 9 shale samples from the Dalong Formation used for high-pressure mercury intrusion experiments were also subjected to nitrogen adsorption experiments. The results indicate that under identical pressure conditions, the nitrogen adsorption capacity of the Longmaxi Formation shale ranges from 13.644 to 21.743 cm^3^/g (average: 16.957 cm^3^/g) (Figure 5a). In comparison, the Dalong Formation shale exhibits a broader range and a slightly higher average adsorption capacity, varying from 9.06 to 30.40 cm^3^/g (average: 19.47 cm^3^/g) (Figure 5b). Regarding pores with diameters smaller than 100 nm, the total pore volume in the Longmaxi Formation shale ranges from 0.0191 to 0.0285 cm^3^/g (average: 0.0240 cm^3^/g) (Figure 5c). For the same nanoscale fraction, the Dalong Formation shale presents a total pore volume ranging from 0.0125 to 0.0434 cm^3^/g, yielding a slightly higher average of 0.0274 cm^3^/g (Figure 5d). For the Longmaxi Formation shale, the specific surface area of pores smaller than 100 nm ranges from 10.772 to 30.076 m^2^/g, with an average value of 19.980 m^2^/g (Figure 5e). For the Dalong Formation shale, the specific surface area of pores smaller than 100 nm ranges from 8.038 to 51.737 m^2^/g, with an average value of 26.492 m^2^/g (Figure 5f).

#### 4.3.3. CO_2_ Adsorption Experiment

**Figure 6:**
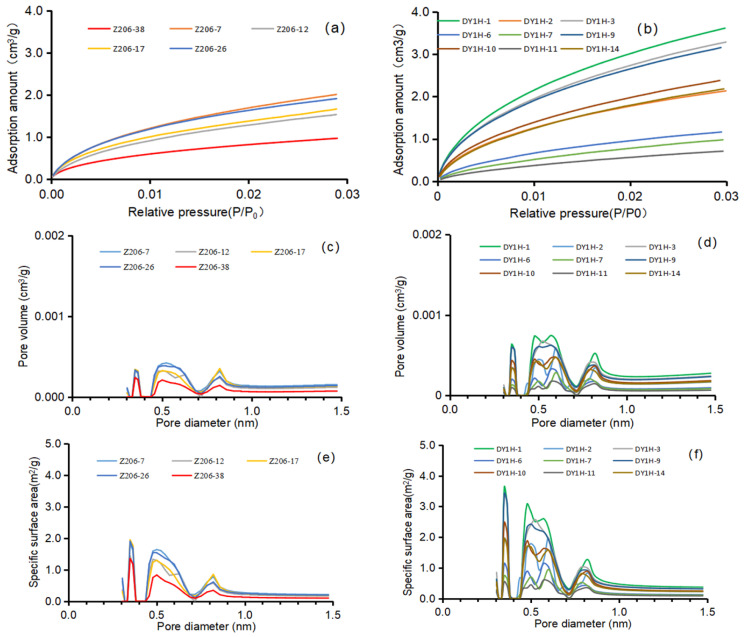
Plates of experimental data for shale CO_2_ Adsorption. (**a**) Adsorption and desorption volumes per unit mass of Longmaxi Formation Shale under different pressure conditions. (**b**) Adsorption and desorption volumes per unit mass of Dalong Formation Shale under different pressure conditions. (**c**) Pore volume of different pore sizes per unit mass of Longmaxi Formation Shale. (**d**) Pore volume of different pore sizes per unit mass of Dalong Formation Shale. (**e**) Specific surface area of different pore sizes per unit mass of Longmaxi Formation Shale. (**f**) Specific surface area of different pore sizes per unit mass of Dalong Formation Shale.

Corresponding CO_2_ adsorption experimental data show that the CO_2_ adsorption capacity of the Longmaxi Formation shale ranges from 0.966 to 2.009 cm^3^/g, with an average value of 1.615 cm^3^/g (Figure 6a). For the Dalong Formation shale, the CO_2_ adsorption capacity ranges from 0.708 to 3.614 cm^3^/g, with an average value of 2.179 cm^3^/g (Figure 6b). The total pore volume of pores smaller than 1.5 nm in the Longmaxi Formation shale is 0.003–0.007 cm^3^/g, with an average value of 0.005 cm^3^/g (Figure 6c). For the Dalong Formation shale, the total pore volume of pores smaller than 1.5 nm is 0.0024–0.0115 cm^3^/g, with an average value of 0.007 cm^3^/g (Figure 6d). The total specific surface area of pores smaller than 1.5 nm in the Longmaxi Formation shale is 10.072–21.098 m^2^/g, with an average value of 16.969 m^2^/g (Figure 6e). For the Dalong Formation shale, the total specific surface area of pores smaller than 1.5 nm is 7.379–37.331 m^2^/g, with an average value of 22.453 m^2^/g (Figure 6f).

## 5. Discussion

### 5.1. Differential Depositional Mechanism and Organic Matter Enrichment Model

#### 5.1.1. Paleostructure

**Figure 7:**
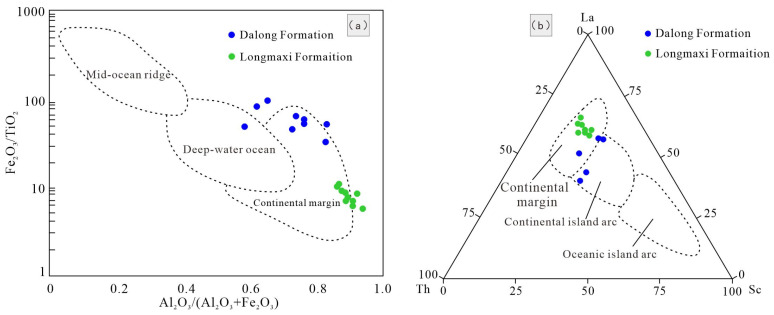
Tectonic setting discrimination diagram of major and trace elements. (**a**) Scatter Plot of Al_2_O_3_/(Al_2_O_3_ + Fe_2_O_3_) and Fe_2_O_3_/TiO_2_. (**b**) Triangular Diagram of La, Th and Sc.

The cross-plot of major element ratios (Al_2_O_3_/(Al_2_O_3_ + Fe_2_O_3_) versus Fe_2_O_3_/TiO_2_) and the La-Th-Sc trace element ternary diagram reveal discernible differences in the paleo-tectonic settings during the deposition of the Lower Silurian Longmaxi Formation and the Upper Permian Dalong Formation (Figure 7a). The tectonic settings for both shale sequences were predominantly characterized by continental margins, aligning well with previous structural geological interpretations. However, the Longmaxi Formation shale data points plot more tightly within the continental margin discrimination field. This positioning indicates a comparatively shallower depositional water mass and a more robust terrigenous input, which is further evidenced by its higher concentrations of the major elements Al_2_O_3_ and Fe_2_O_3_, along with a more pronounced enrichment of the trace element La (Figure 7b).

#### 5.1.2. Paleoclimate

**Figure 8:**
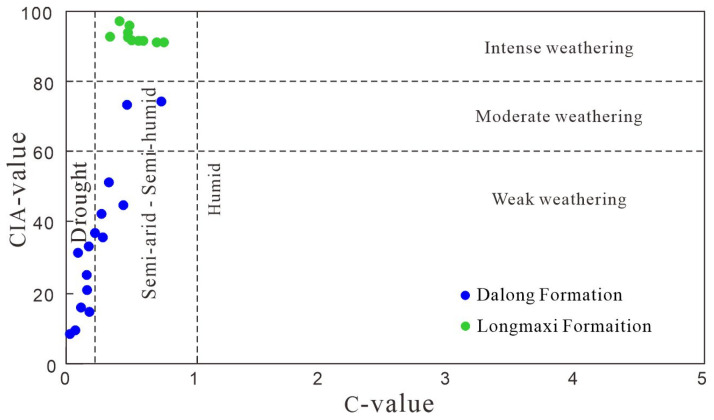
Paleoclimatic discrimination diagram of major and trace elements.

Typically, the Chemical Index of Alteration (CIA, calculated as [Al_2_O_3_/(Al_2_O_3_ + CaO* + Na_2_O + K_2_O)] × 100) and the C-value (Σ(Fe + Mn + Cr + Ni + V+Co)/Σ(Ca + Mg + K+Na + Sr + Ba)) are widely utilized to comprehensively evaluate paleoclimatic conditions (humid versus arid) and the degree of chemical weathering during shale deposition [21]. Calculations and data plots of these indices for both shale sequences (Figure 8) indicate that the CIA values of the Longmaxi Formation shale range from 90.80 to 96.69 (average: 92.44), whereas those of the Dalong Formation exhibit a notably lower range of 8.24 to 73.99 (average: 33.18). Similarly, the C-values of the Longmaxi Formation range from 0.35 to 0.76 (average: 0.54), compared to 0.03 to 0.739 (average: 0.24) for the Dalong Formation. The significantly higher CIA and C-values in the Longmaxi Formation suggest that it was deposited predominantly under a humid paleoclimate, characterized by moderate to intense chemical weathering. Conversely, the Dalong Formation was primarily deposited under arid to semi-arid/semi-humid conditions, reflecting a relatively weak degree of weathering.

#### 5.1.3. Redox Conditions

**Figure 9:**
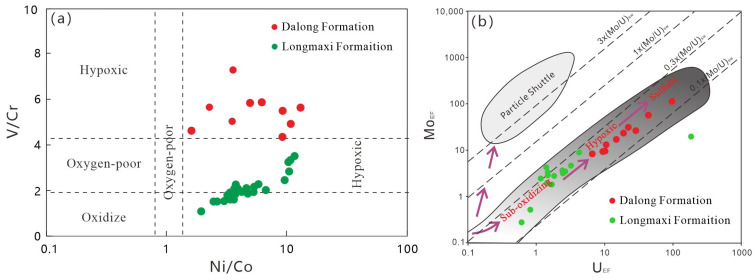
Shale bimetallic element ratio diagram and Mo_EF_–U_EF_ diagram. (**a**) Scatter Plot of Ni/Co and V/Cr. (**b**) Scatter Plot of MoEF and UEF.

The redox conditions of marine water columns govern the differential enrichment of redox-sensitive elements (such as V, Cr, and U) as well as the preservation of organic matter, rendering it a persistent focal point in environmental geochemistry research. Traditionally, higher V/Cr and Ni/Co ratios indicate a greater degree of anoxia in the bottom water [22]. During the deposition of the Early Silurian Longmaxi Formation, the V/Cr ratios ranged from 0.87 to 2.60 (average: 1.45), while the Ni/Co ratios ranged from 3.38 to 9.27 (average: 5.72). In contrast, the Late Permian Dalong Formation exhibited V/Cr ratios between 0.06 and 4.11 (average: 0.26) and Ni/Co ratios ranging from 0.60 to 12.76 (average: 7.23). Despite some internal variations, the bimetallic element ratio data collectively suggest that the degree of anoxia during the Longmaxi Formation shale deposition was significantly weaker than that of the Dalong Formation (Figure 9a). Similarly, the redox conditions of bottom water can be determined based on the Mo_EF_-U_EF_ diagram [23]. The Mo_EF_-U_EF_ diagram (Figure 9b) for the Dalong Formation and Longmaxi Formation shales shows that Mo_EF_ and U_EF_ exhibit a significant positive correlation in both formations, with data points mainly distributed in the anoxic-sulfidic zone, indicating that both formations were deposited under strongly reducing conditions. Overall, the Mo_EF_ and U_EF_ values of the Dalong Formation shale are distinctly higher than those of the Longmaxi Formation shale, reflecting that the reduction intensity during the Late Permian deposition was generally stronger than that during the Early Silurian Longmaxi Formation deposition.

#### 5.1.4. Terrestrial Input and Restriction Degree

**Figure 10:**
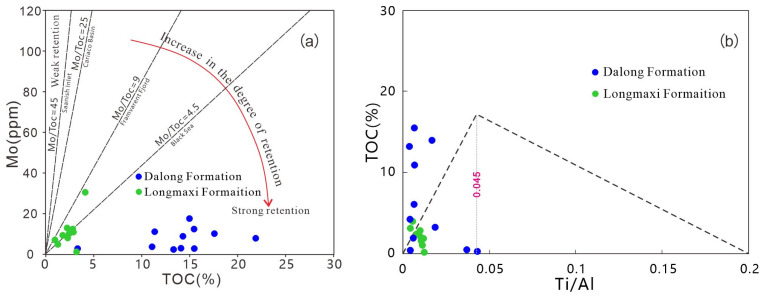
Discrimination diagram of water mass restriction (**a**) and terrestrial input degree (**b**).

The preservation quality of organic matter in shales is often related to the degree of water mass restriction in sedimentary basins. Generally, the correlation between Mo content and TOC content in shales can be used to determine the degree of water mass restriction [24]. A higher Mo/TOC ratio indicates a lower degree of water restriction. The Mo/TOC ratio of the Longmaxi Formation shale ranges from 0 to 9, whereas that of the Dalong Formation is all less than 4.5. The Dalong Formation shale was deposited in a sulfidic water environment (Figure 10a), suggesting that both the Longmaxi and Dalong Formation shales were formed under strongly restricted conditions, with a higher sulfidation degree in the Dalong Formation.

Meanwhile, the enrichment degree of organic matter in sedimentary basins is affected by terrigenous clastic input and sedimentation rate (SR). The Ti/Al ratio can reflect the magnitude of terrigenous clastic input and sedimentation rate: a higher Ti/Al ratio indicates greater terrigenous input and a faster sedimentation rate. The TOC-Ti/Al cross-plot results for the Longmaxi Formation and Dalong Formation shales (Figure 10b) show that the Ti/Al values of the Dalong Formation shale are generally less than 0.02, reflecting weak terrigenous input and a low sedimentation rate at that time. Meanwhile, both the Longmaxi and Dalong Formation shales exhibit a trend where TOC values gradually increase with increasing sedimentation rate and terrigenous input. This indicates that terrigenous input was generally weak during shale deposition. Appropriate terrigenous input can provide nutrients for plankton, promote organic matter production, which can then be effectively preserved under strong reducing conditions.

#### 5.1.5. Paleoproductivity

Paleoproductivity within various sedimentary basins is comprehensively governed by a combination of factors, including paleotectonic settings, paleoclimate, water-column redox conditions, and terrigenous input. Consequently, accurately and effectively estimating paleoproductivity remains a critical challenge in petroleum geochemistry. Historically, total organic carbon (TOC), biogenic barium (Ba_bio_), and organic phosphorus (P_org_) have been widely employed as primary proxies to indicate paleoproductivity during shale deposition. However, the application of these indices is frequently complicated by confounding factors and overlapping signals [7]. For instance, only 0.1% to 10% of surface-water organic matter survives oxidative degradation in the water column to be ultimately preserved in the sediment. Furthermore, terrigenous input can act as a double-edged sword: it may dilute the concentration of marine organic matter, but it can also supply essential nutrients that fuel marine phytoplankton blooms [22]. Additionally, under strongly reducing conditions, elements such as phosphorus (P) and barium (Ba) are susceptible to intense dissolution. This mobilization can consequently lead to a significant underestimation of the actual paleoproductivity. To address this issue, scholars usually use the normalized enrichment factors of nutrient elements (Cu, Ni, Zn) to reflect paleoproductivity in seawater under reducing conditions [25]. The element enrichment factor X_EF_ is calculated by the formula: X_EF_ = [(X/Al)sample/(X/Al)_PASS_], where (X/Al)_PASS_ is the elemental ratio of Post-Archean Australian Shale. X_EF_ > 1 indicates enrichment relative to PASS, and depletion vice versa. In terms of Cu_EF_, Ni_EF_, and Zn_EF_: For the Dalong Formation shale, Cu_EF_ ranges from 1.41 to 10.69 ppm with an average of 5.50 ppm; Ni_EF_ and Zn_EF_ range from 1.34 to 9.55 ppm and 1.13 to 5.35 ppm, with averages of 4.52 ppm and 3.16 ppm, respectively. For the Longmaxi Formation shale, Cu_EF_ ranges from 0.33 to 0.59 ppm with an average of 0.44 ppm; Ni_EF_ and Zn_EF_ range from 0.18 to 0.44 ppm and 0.09 to 1.28 ppm, with averages of 0.33 ppm and 0.41 ppm, respectively. This indicates that paleoproductivity during the Late Permian deposition was significantly higher than that during the Early Silurian.

#### 5.1.6. Differential Enrichment Model of Organic Matter

**Figure 11:**
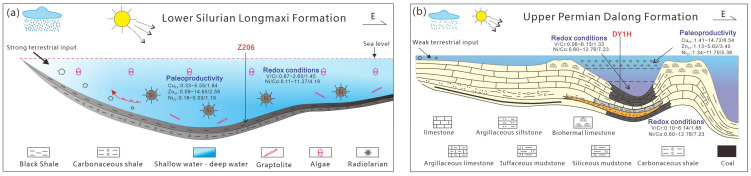
Diagram of Differential Enrichment Patterns of Organic Matter in the Longmaxi (**a**) and Dalong (**b**) Formations.

**Figure 12:**
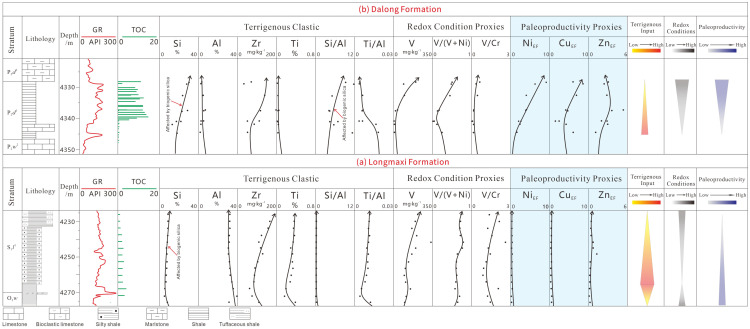
Paleoenvironmental Parameter Comparison Diagram of the Longmaxi Formation (**a**) and Dalong Formation (**b**) Shales.

Deposited in a shelf setting, the Longmaxi Formation is characterized by a paleoenvironmental signature of “high terrigenous input, moderate-to-high reducing conditions, and low paleoproductivity.” It exhibits a relatively high degree of marine openness, with the shale predominantly composed of quartz and clay minerals (Figure 11a). In contrast, the Dalong Formation was deposited in an intra-platform basin setting. Its overall paleoenvironment is characterized by “low terrigenous input, highly reducing conditions, and high paleoproductivity.” The depositional water mass was relatively restricted, and the resulting shale is rich in quartz and carbonate minerals, generally exhibiting higher TOC values (Figure 11b). Furthermore, the vertical coupling relationship diagram among terrigenous input, redox conditions, and paleoproductivity for the Longmaxi Formation shale in Well Z206 and the Dalong Formation shale in Well D201 (Figure 12) reveals distinct evolutionary pathways. The Early Silurian depositional paleoenvironment was relatively stable, exhibiting minor vertical fluctuations in elemental proxies (Figure 12a). Overall, it displays a trend of weak terrigenous input in the early stage that subsequently strengthened in the late stage, accompanied by an upward-decreasing trend in both the degree of anoxia and paleoproductivity. Conversely, profoundly influenced by tectonic extension during the Late Permian, the depositional setting gradually evolved from an early carbonate ramp to an intra-platform basin. Consequently, the Late Permian paleoenvironment exhibits distinct vertical variations from the early to late stages, characterized by a weakening of terrigenous input, an intensification of reducing conditions, and a marked enhancement in paleoproductivity (Figure 12b).

### 5.2. Reservoir Differential Characteristics Under Differential Deposition

Differential sedimentation leads to variations in the mineralogical composition of shales. Consequently, different mineral assemblages exhibit profoundly distinct reservoir spaces and pore structures following diagenetic modifications [5,26]. To better characterize the pore structure of these shale reservoirs, a comprehensive approach integrating high-pressure mercury injection (HPMI), low-temperature nitrogen adsorption, and CO_2_ adsorption was employed to elucidate the developmental characteristics and distribution patterns of pores across multiple scales. Specifically, macropores (>50 nm) were characterized using HPMI data, mesopores (2–50 nm) were evaluated using nitrogen adsorption data, and micropores (<2 nm) were jointly characterized by combining CO_2_ and nitrogen adsorption analyses.

#### 5.2.1. Full Pore Size Characterization

**Figure 13:**
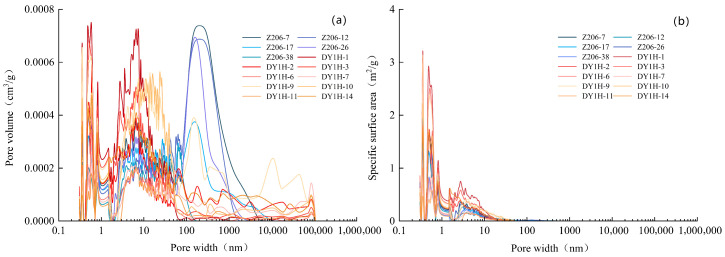
Pore structure characteristics of full pore size in different shales. (**a**) Pore volume distribution of different pore sizes per unit mass of shale. (**b**) Specific surface area distribution of different pore sizes per unit mass of shale.

By characterizing the nanoscale pores across different size ranges, distinct differences in the nano-reservoir structures between the Longmaxi and Dalong formation shales become clearly evident. Although the total nanoscale pore volume for both the Dalong and Longmaxi formation shales is approximately 0.03 cm^3^/g, their detailed pore volume distributions differ. In both formations, the nanoscale pore volume is predominantly contributed by mesopores, accounting for approximately 65% (Table 1), followed by micropores (20–30%). However, nanoscale macropores (>50 nm) are significantly more developed in the Longmaxi Formation shale, comprising 17.02% of its total pore volume, whereas they account for only 3.53% in the Dalong Formation shale (Figure 13a). Correspondingly, the specific surface areas of both the Dalong and Longmaxi formation shales are primarily provided by micropores and mesopores (Table 1). Nevertheless, because these micropores and mesopores are relatively more developed in the Dalong Formation, the specific surface area contributed by pores within the same size range is notably larger. Consequently, the Dalong Formation shale exhibits a higher average nanoscale specific surface area of 41.88 m^2^/g, whereas the Longmaxi Formation shale averages only 30.03 m^2^/g (Figure 13b).

#### 5.2.2. Microscopic Differential Laws and Controlling Factors of Reservoirs

**Figure 14:**
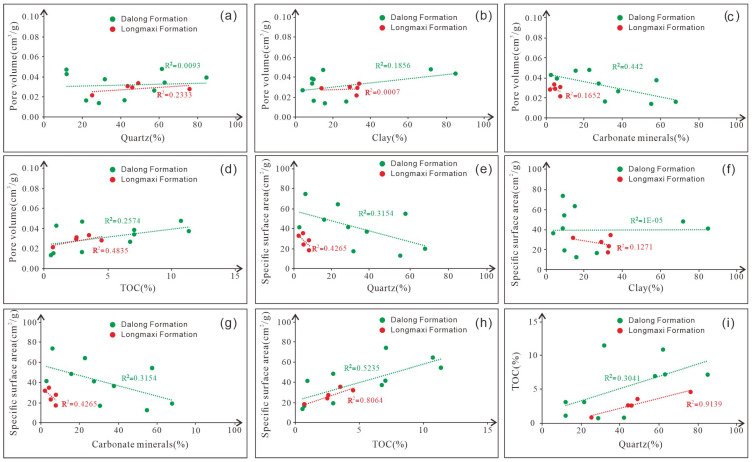
Relationship between pore size & specific surface area of different nanoscale pores and TOC in shale. (**a**) Relationship between quartz content and pore volume. (**b**) Relationship between clay content and pore volume. (**c**) Relationship between carbonate minerals content and pore volume. (**d**) Relationship between TOC content and pore volume. (**e**) Relationship between quartz content and Specific surface area volume. (**f**) Relationship between clay content and Specific surface area volume. (**g**) Relationship between carbonate minerals content and Specific surface area volume. (**h**) Relationship between TOC content and Specific surface area volume. (**i**) Relationship between quartz and TOC content.

Undoubtedly, the depositional paleoenvironment and sedimentary facies dictate the primary mineral composition and TOC of shales. However, following diagenetic evolution, it is these different minerals and organic matter that ultimately control the pore types and structures within the reservoir [7,9,26]. The mineral composition and TOC of the Dalong Formation shale differ significantly from those of the Longmaxi Formation. Compared to the latter, the Dalong Formation is typically characterized by a “high carbonate mineral content and high TOC” signature. By establishing the correlations between the nanoscale pore volume/specific surface area and various minerals/TOC for both the Longmaxi and Dalong formation shales (Figure 14), distinct controlling mechanisms are revealed. Quartz, clay minerals, and TOC exhibit a pronounced promotional effect on nanoscale pore volume, whereas carbonate mineral content shows a negative correlation with pore volume (Figure 14a–d). Correspondingly, quartz and carbonate minerals exhibit negative correlations with nanoscale specific surface area, while clay minerals and TOC demonstrate positive correlations (Figure 14e–h). In organic-rich shales, quartz content generally exhibits a positive correlation with TOC (Figure 14i). Previous studies have long confirmed that a portion of the silica in marine shales of southern China is of deep-water biogenic origin (e.g., radiolarians and graptolites). Higher organic matter content in the shale corresponds to relatively more developed nanoscale micropores and mesopores, leading to distinct increases in both pore volume and specific surface area (Figure 14d,h). In addition to biogenic quartz, these shales contain abundant terrigenous and authigenic quartz grains, which are unfavorable for the development of nanoscale pores. Consequently, total quartz content exhibits only a weak positive correlation with nanoscale pore volume (Figure 14a) and a distinct negative correlation with specific surface area (Figure 14e). Furthermore, higher carbonate mineral content leads to a relative decrease (dilution) in quartz and organic matter contents, thereby causing a certain degree of reduction in both pore volume and specific surface area (Figure 14c,g). Finally, while clay minerals exert a certain promotional effect on the development of shale nanoscale pores, they have little influence on the specific surface area.

The Dalong Formation was deposited within an intra-platform basin setting, characterized by minimal terrigenous input, highly reducing conditions, and robust paleoproductivity (Figure 11). Consequently, the resulting shales exhibit notably high carbonate mineral and TOC contents. Driven by these compositional characteristics, the diagenetic dissolution of carbonate minerals in the Dalong Formation promotes the relative development of micrometer-scale pores larger than 100 μm (Figure 4d). Within the nanoscale spectrum, micropores and mesopores are more extensively developed, accounting for a larger relative proportion and yielding a specific surface area significantly greater than that of the Longmaxi Formation (Table 1). In contrast, the Longmaxi Formation was deposited in a continental shelf environment, featuring strong terrigenous input, less intense reducing conditions, and relatively weaker paleoproductivity (Figure 11). Consequently, these shales contain higher amounts of terrigenous quartz and relatively lower TOC. While their micrometer-scale pores are less developed compared to those of the Dalong Formation (Figure 4d), their nanoscale macropores are significantly more developed than those in the Dalong Formation (Figure 13). Based on the comprehensive research presented above, the microscopic reservoir characteristics of the organic-rich shales from the Early Silurian Longmaxi and Late Permian Dalong formations in the Sichuan Basin are comprehensively controlled by their respective depositional conditions. Organic-rich intervals with high TOC exhibit fundamentally superior reservoir properties. Ultimately, the high-quality shales situated in the lower part of the Longmaxi Formation and the upper part of the Dalong Formation are identified as the primary sweet spot targets for future shale gas exploration and development.

## 6. Conclusions

(1) The Lower Silurian Longmaxi Formation shale represents a continental shelf facies deposit, with its mineralogy dominated by quartz and clay minerals. Conversely, the Upper Permian Dalong Formation was deposited in an intra-platform basin setting, primarily consisting of quartz and carbonate minerals. Compared to the Longmaxi Formation, the depositional period of the Dalong Formation was characterized by weaker terrigenous input, more intensely reducing conditions, and higher paleoproductivity, ultimately yielding higher TOC values.
(2) Carbonate mineral dissolution can promote the development of micron-scale pores in shales. Kerogen nanopores and bitumen organic pores after organic matter thermal evolution significantly contribute to the formation of nano-scale micropores and mesopores. In comparison, macro-nanopores are better developed in the Longmaxi Formation shale. The Dalong Formation shale is rich in carbonate minerals and organic matter, so micron-scale pores, nano-scale micropores and mesopores are more developed, with larger specific surface area and stronger shale adsorption capacity.
(3) The development degree and adsorption capacity of nanopores in shale reservoirs are mainly affected and restricted by organic matter; therefore, the organic-rich shale intervals represent the critical “sweet spot” target layers for future shale gas exploration and development within both the Longmaxi and Dalong formations.

## Figures and Tables

**Table 1:** Parameters for Nanopore Volume and Specific Surface Area of Different Pore Diameters in Longmaxi Formation and Dalong Formation Shales.

Stratum	Micropore (<2 nm)				Mesopore (2~50 nm)				Macropore (>50 nm)			
	Pore Volume (cm^3^/g)	Proportion (%)	Specific Surface Area (m^2^/g)	Proportion (%)	Pore Volume (cm^3^/g)	Proportion (%)	Specific Surface Area (m^2^/g)	Proportion (%)	Pore Volume (cm^3^/g)	Proportion (%)	Specific Surface Area (m^2^/g)	Proportion (%)
Dalong Formation	0.0087	27.66	28.033	69.26	0.0218	68.81	12.442	30.74	0.0011	3.53	0.000005	0.00
Longmaxi Formation	0.0054	19.15	17.488	64.91	0.0180	63.83	9.234	34.27	0.0048	17.02	0.222200	0.82

## Data Availability

The original contributions presented in this study are included in the article. Further inquiries can be directed to the corresponding authors.
